# The Prognostic Significance of Hsp70 in Patients with Colorectal Cancer Patients: A PRISMA-Compliant Meta-Analysis

**DOI:** 10.1155/2021/5526327

**Published:** 2021-04-16

**Authors:** Guangyu Gao, Songtao Liu, Zhen Yao, Yanyan Zhan, Wenyue Chen, Yulong Liu

**Affiliations:** ^1^Department of Nuclear Accident Medical Emergency, The Second Affiliated Hospital of Soochow University, Suzhou 215004, China; ^2^Department of Ultrasound, The Second Affiliated Hospital of Soochow University, Suzhou 215004, China; ^3^State Key Laboratory of Radiation Medicine and Protection, School of Radiation Medicine and Protection, Soochow University, Suzhou 215123, China; ^4^Collaborative Innovation Center of Radiological Medicine of Jiangsu Higher Education Institutions, Suzhou 215123, China

## Abstract

**Background:**

Hsp70 (heat shock protein 70) plays a key role in carcinogenesis and cancer progression. However, the relationship between the Hsp70 expression level and the colorectal cancer patient survival is unknown. This study is aimed at investigating the relationship between Hsp70 and the prognosis of colorectal carcinoma patients.

**Methods:**

PubMed, Web of Science, and Embase were used for systematic computer literature retrieval. Stata SE14.0 software was used for quantitative meta-analysis. Besides, data was extracted from selected articles. Relationships between Hsp70 expression level and prognosis were further studied. The hazard ratios (HRs) and 95% confidence intervals (95% CIs) were also computed.

**Results:**

A total of 11 potentially eligible studies with 2269 patients were identified in 10 tumors from PubMed, Web of Science, and Embase. Hsp70 overexpression was associated with poor overall survival (OS) and disease-free survival (DFS) in colorectal carcinoma patients (HRs, 0.65 (95% CI: 0.52-0.78) and 0.77 (95% CI: 0.23-1.32), respectively).

**Conclusions:**

Hsp70 overexpression can predict poor survival in colorectal cancer patients.

## 1. Introduction

Colorectal cancer (CRC) is a serious health problem worldwide. The response and overall survival of advanced colorectal cancer patients are very poor compared with the early stage. While colorectal cancer can be treated through surgery, tumor recurrence develops in about 25% to 40% of patients [1]. Surgical tumor resection is the major treatment method for local advanced colorectal cancer. However, there is no effective treatment for metastatic tumors, especially those that cannot be surgically removed and those with poor chemotherapy and radiotherapy effects [2].

Hsps are well-maintained molecules overexpressed in cells subjected to various stress stimuli (heat shock or disruption of homeostasis). Hsps act as molecular chaperones assisting protein folding in normal metabolic conditions and promoting protein repair and stabilization during molecular stress [3]. Hsps are essential in cellular defense against carcinogenesis [4]. Their tumorigenesis role includes oncogenic protein stabilization, programmed cell death, replicative senescence inhibition, tumor angiogenesis induction, invasion, and metastasis activation [5]. Interestingly, most Hsp70 expression studies are based on colorectal cancer cell lines and xenografts and not actual biopsy samples [6].

Studies have shown that Hsp70 expression is upregulated in various carcinoma tissues and could be a potential biomarker [7]. Besides, previous studies have reported that Hsp70 overexpression is associated with poor survival in some cancers, including breast carcinoma [8–10], esophageal adenocarcinoma [11], non-small-cell lung cancer [12], prostate cancer [13, 14], gastric cancer [15, 16], leukemia [17], hepatocellular carcinoma [18, 19], pancreatic cancer [20], and colon cancer [21–23]. However, no study has reported the relationship between the Hsp70 expression level and prognosis of colorectal cancer patients. Therefore, in this study, the relationship between Hsp70 and the prognosis of colorectal carcinoma patients was investigated.

## 2. Methods

### 2.1. Literature Retrieval

Studies published between July 9, 1989 and July 9, 2020 were downloaded from PubMed, Web of Science, and Embase. Text word and MeSH strategy were used for the retrieval. The following terms were used: heat shock protein 70 OR Hsp70 AND colorectal AND cancer OR carcinoma OR malignant OR neoplasm OR tumor. Besides, the reference lists of the retrieved studies were manually screened for additional literature retrieval.

### 2.2. Selection Criteria

Two researchers independently extracted the studies. The inclusion criteria were as follows: (a) studies showing the relationship between the Hsp70 expression level and OS or DFS in colorectal carcinoma, (b) studies describing the relationship between clinicopathological parameters and Hsp70 expression levels in human carcinoma tissues, (c) studies grouping patients (two groups) based on Hsp70 expression level, and (d) if hazard ratios and 95% confidence intervals could be calculated. The exclusion criteria were as follows: (a) studies with insufficient information to compute hazard ratios and 95% confidence intervals or (b) letters, case reports, expert opinions, and reviews or (c) *in vitro* or animal studies. Besides, only English researches were included.

### 2.3. Data Extraction

Two authors (GY and ZY) extracted data from identified articles that complied with the standards, and a third researcher resolved different opinions. The first author, country, year of publication, tumor type, sample size, study design, cut-off value, follow-up time, methods used, and the outcome were recorded. Hazard ratios with 95% confidence intervals for overall survival or disease-free survival were also determined. Engauge Digitizer version 4.1 was used to calculate prognosis after drawing the Kaplan-Meier curves [24].

### 2.4. Statistical Methods

The prognostic value of the Hsp70 expression level in colorectal cancer patients was determined by estimating the hazard ratios between the upregulated and downregulated Hsp70 tissue groups for overall survival or disease-free survival. The 95% confidence intervals and the heterogeneity were also calculated and evaluated based on *P* value and *I*^2^. Furthermore, *I*^2^ > 50% indicated significant heterogeneity. The random-effects model was used to determine the total hazard ratios. A fixed-effects model was used when *I*^2^ ≤ 50% (moderate heterogeneity). The Stata 14.0 software was used for all data analyses.

## 3. Results

### 3.1. Included Studies and Characteristics

**Figure 1:**
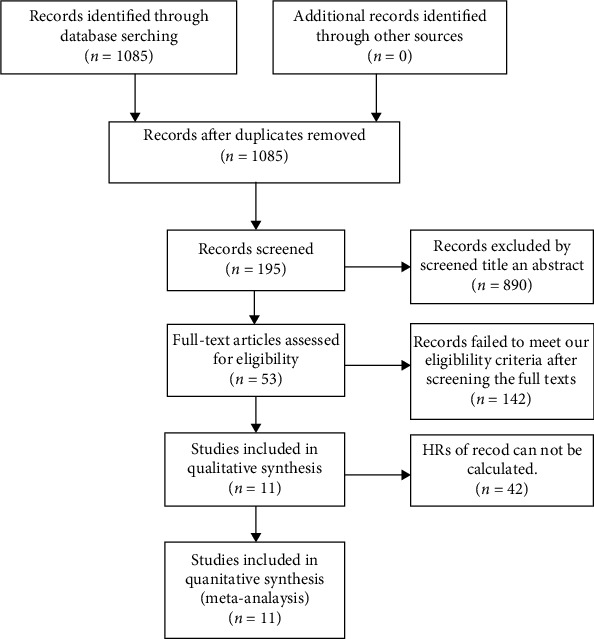
The flow chart of the selection process in the meta-analysis.

The research procedure is shown in Figure 1. A total of 1085 articles were selected after screening Web of Science, Embase, and PubMed. A total of 890 studies were excluded after the title and abstract search. Moreover, 142 articles were excluded since they did not meet the inclusion criteria. Another 42 records had insufficient data for HR calculation. Finally, 11 articles with 2269 patients were used [21–23, 25–32]. The 11 articles had a sample size of 167 to 256 (average 206) (Table 1). Besides, the 11 articles were published between 2009 and 2020 from various countries, including Hungary (4), Korea (2), and Britain, Greece, and Jordan (1 each). Most articles used immunohistochemistry (IHC) to determine the Hsp70 expression level.

### 3.2. High Hsp70 Expression Is Associated with Poor OS

**Figure 2:**
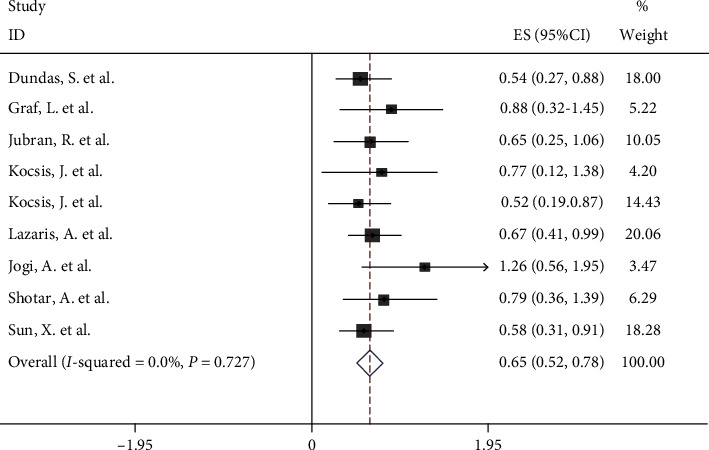
The relationship between Hsp70 expression and overall survival (OS) in human colorectal cancer.

Systematic meta-analysis was used to assess the relationship between the Hsp70 expression level and OS in colorectal cancer patients. Meta-analysis of nine articles with 1917 colorectal cancer patients showed that Hsp70 expression was significantly associated with OS (HR 0.65; 95% CI: 0.52-0.78; Figure 2), indicating that Hsp70 overexpression is associated with poor survival. A fixed-effects model was used since no significant heterogeneity was found (*I*^2^ = 0%) in the identified studies.

### 3.3. High Hsp70 Expression Is Associated with Poor DFS

**Figure 3:**
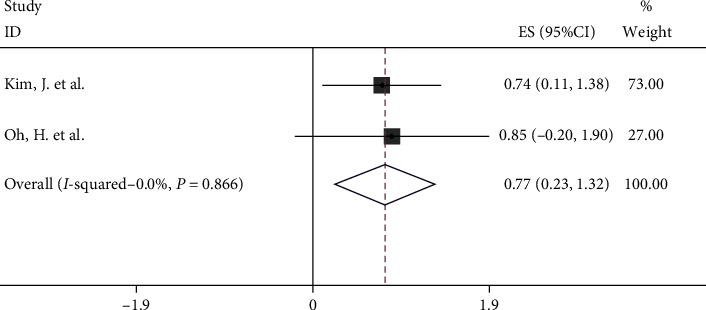
Forest plot indicating the association between Hsp70 expression and DFS.

The relationship between Hsp70 expression and disease-free survival of 352 cancer patients was also investigated (Figure 3). A fixed-effects model was used since there was no significant heterogeneity (*I*^2^ = 0). The upregulated Hsp70 was associated with poor disease-free survival (HR = 0.77, 95% CI: 0.23-1.32).

### 3.4. Publication Bias

**Figure 4:**
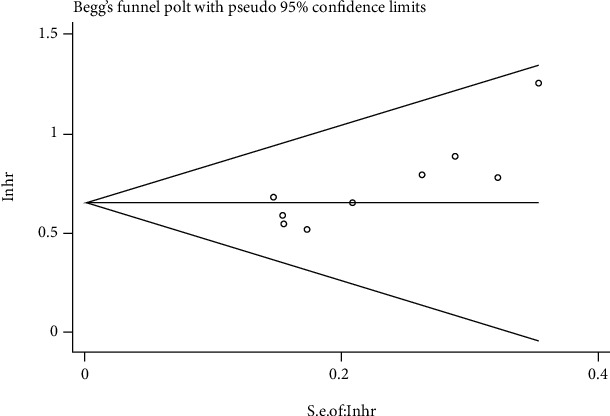
Begg and Egger tests of OS.

**Figure 5:**
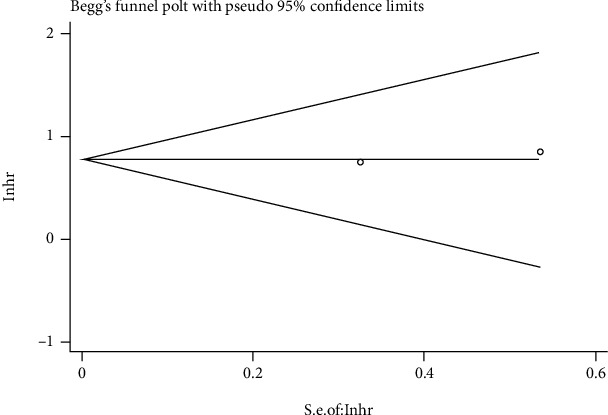
Begg and Egger tests of DFS.

**Figure 6:**
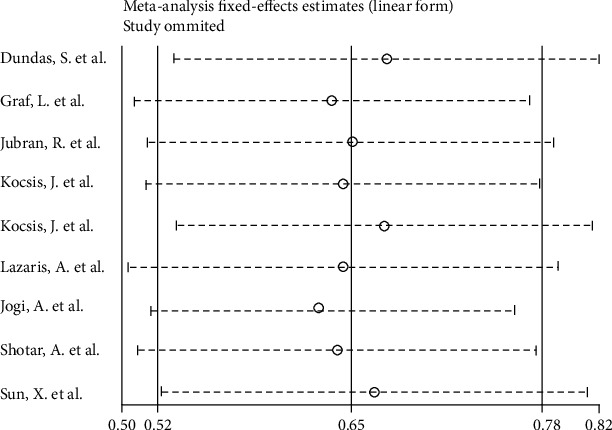
Sensitivity analysis evaluating the impact of each research.

Begg's and Egger's tests were used to evaluate the publication bias via funnel plot symmetry estimation. There was no significant asymmetry of the funnel plots. Therefore, this study has no significant publication bias [33] (Figures 4 and 5). A sensitivity analysis was also conducted to verify the credibility of hazard ratios for overall survival. There were no significant impacts on HRs after excluding any one study, suggesting result reliability (Figure 6).

## 4. Discussion

In recent years, potential prognostic biomarkers have been identified to improve the efficacy and survival rate. However, the prognosis of most cancers is relatively low. Therefore, high/positive tissue Hsp70 expression could be associated with the prognosis of colorectal carcinoma patients. Furthermore, previous studies have concluded that Hsp70 expression has biological and clinical significance. However, various cancers have different results. Hsp70 is significantly upregulated in gastric, breast, and prostate carcinoma compared with the normal tissues. In contrast, some articles have indicated that Hsp70 is significantly downregulated in cancer tissues compared with normal tissues in malignant melanoma, colon cancer, and urothelial bladder cancer. The different results could be due to the various interactions between Hsp70 and other human gene products in different organs. Recent articles have verified that Hsp70 overexpression is associated with poor survival in breast cancer, colorectal cancer, melanoma, gastric cancer, hepatocellular carcinoma, non-small-cell lung cancer, ovarian cancer, and testicular cancer. Besides, Hsp70 overexpression is not associated with esophageal adenocarcinoma and esophageal squamous cell cancer, while it is associated with better survival in urinary bladder cancer and pancreatic cancer. Sun et al. reported no significant relationships between Hsp with sex or age of patients, tumor site, Duke's stage, growth pattern, or differentiation [34]. Shotar also reported that there is no significant relationship between the HSP70 and p53 expressions [30]. Similarly, Kocsis et al. reported that mortalin blood analysis, mortalin, and sHSP70 have a high prognostic value at the TNM stage and can identify colorectal cancer patients at high risk of poor survival [29]. Oh et al. also reported that the dominant intensity score-based Hsp70 IHC interpretation can be an effective method for MSI-H-prognostic stratification (a high level of microsatellite instability) in colorectal cancer [22]. In this study, the prognostic value and clinicopathologic significance of Hsp70 in colorectal carcinoma patients were assessed. This study can help understand the tumor generation process and the treatment value of Hsp70 in colorectal cancer. The meta-analysis included 2269 tumor patients from 11 articles (Hungary (4), Korea (2), and Britain, Greece, and Jordan (1 each)). A fixed-effects model was used for heterogeneity examination. The combined HRs indicated that Hsp70 overexpression was associated with the poor prognosis of colorectal carcinoma patients. Hsp70 overexpression was also related to poor DFS. However, a large study should be conducted to verify these results.

Notably, this study has several limitations. First, some survival statistics from digital computer technology should be carefully considered. Besides, non-English articles were excluded. Second, only 11 articles with 2269 patients were included. There were no enough articles for subgroup analyses on disease-free survival. Third, different studies had different definitions of Hsp70 expression. Therefore, more researches are needed for more inclusive conclusions.

## 5. Conclusion

Hsp70 overexpression is significantly associated with poor overall survival and disease-free survival in colorectal carcinoma patients. Several such studies can provide mechanistic, pathophysiological, and epidemiological evidence complementing the findings of the current clinical trials on Hsp70 inhibitors.

## Figures and Tables

**Table 1:** Main characteristics of the 11 studies in the meta-analysis.

Study	Country	Year	Sample	Study design	Sizes	Cut-off value	Gender (female/male)	Follow-up time (month)	Tumor stage (I/II/III/IV)	Outcome	Method	NOS
Dundas, S. et al.	Britain	2005	CRC	R	231	25%	NA	0-120	NA	OS	IHC	7
Graf, L. et al.	Hungary	2018	CRC	R	232	20%	112/120	0-60	NA	OS	IHC	7
Jubran, R. et al.	Hungary	2017	CRC	R	227	NR	121/106	0-60	30/40/60/97	OS	IHC	8
Kocsis, J. et al.	Hungary	2009	CRC	R	178	NR	89/88	33 (24-44)	25/26/60/67	OS	IHC	9
Kocsis, J. et al.	Hungary	2010	CRC	R	254	NR	NA	33.0 (23.5–43.5)	80/60/110/4	OS	IHC	8
Lazaris, A. et al.	Greece	1997	CRC	R	128	25%	NA	44.76 (0-222.36)	NA	OS	IHC	9
Jogi, A. et al.	Sweden	2009	CRC	R	196	25%	NA	0-60	NA	OS	IHC	8
Shotar, A. et al.	Jordan	2005	CRC	R	215	20%	112/103	6.88 (0.64-17.05)	40/32/79/64	OS	IHC	8
Sun, X. et al.	Sweden	1997	CRC	R	256	20%	122/134	0-156	NA	OS	IHC	7
Kim, J. et al.	Korea	2014	CRC	R	167	25%	NA	29 (0-120)	NA	DFS	IHC	9
Oh, H. et al.	Korea	2017	CRC	R	185	NR	90/95	51.1 (3.1-57.1)	NA	DFS	IHC	9

Abbreviation: CRC: colorectal cancer; R: retrospective analysis; NR: not report; OS: overall survival; DFS: disease-free survival; IHC: immunohistochemistry; NA: not available; NOS: Newcastle-Ottawa Scale.

## Data Availability

All data included in this study are available from the corresponding author upon request.

## Abbreviations

Hsp70: Heat shock protein 70
CI: Confidence intervention
Embase: Excerpta Medica database
HR: Hazard ratio
OS: Overall survival
DFS: Disease-free survival
CRC: Colorectal cancer.
